# Social enterprises’ impact on older people’s health and wellbeing: exploring Scottish experiences

**DOI:** 10.1093/heapro/daz102

**Published:** 2019-10-09

**Authors:** Fiona Henderson, Artur Steiner, Micaela Mazzei, Catherine Docherty

**Affiliations:** 1 Research and Innovation Office; 2 Yunus Centre for Social Business and Health, Glasgow Caledonian University, Cowcaddens Road, Glasgow G4 0BA, UK

**Keywords:** social enterprise, older people, health

## Abstract

The global aging demographic is putting pressure on state-delivered health and social care services. As the austerity agenda in the UK cuts state-funded service provision for older people despite increasing demand, social enterprise has become a politically and economically attractive model for the sustainable delivery of some public services. Yet little is known about the impact of social enterprise on the health and wellbeing of older people. In this paper we address this gap in understanding and consider social enterprise activities as complex public health-promoting interventions. Our study aimed to understand *what* impact social enterprise activities had on the health and wellbeing of participants aged over 50, and also *how* that impact was created. To achieve this, we conducted qualitative semi-structured interviews with a sample (*n* = 43) of staff, volunteers, clients and carers aged over 50 who were involved in activities delivered by three social enterprises. Using a thematic analysis to explore manifest and latent themes, two antecedents of subjective younger age emerged explaining *how* benefit was created, namely *downward social comparison* and *identity*. The social enterprise activities we studied benefited participants' health and wellbeing, impacting positively on participants' sense of purpose, social support, connectedness and inclusion. These health and wellbeing benefits can be considered as outcomes of complex public health interventions for older people, and we relate these outcomes to beneficial conditions within the intermediary social determinants of health. We conclude by discussing the future impact of social enterprise activities and current UK policy on the structural determinants of health.

## INTRODUCTION

Social enterprises trade goods and services commercially for explicitly social purposes and offer potentially innovative local solutions to social challenges (Gras and Mendoza-Abarca, 2014; Mazzei and Roy, 2017; Henderson *et al.*, 2018). The social missions of social enterprises span a wide range of tangible social needs, such as housing (Manzi and Morrison, 2018) and employment/skills development (Gidron and Monnickendam‐Givon, 2017), but also have less tangible missions related to social connectedness, creative expression, confidence building and/or creating spaces for communities (Eversole *et al.*, 2014; Sacchetti and Campbell, 2014). However disparate these missions are, all activities provided by social enterprises can be conceptualized as addressing vulnerabilities, such as unemployment, poverty, rural isolation and frailty (Haugh and Kitson, 2007; Donaldson *et al.*, 2014). These vulnerabilities map very closely onto widely agreed models of the social determinants of health (Solar and Irwin, 2010; Marmot *et al.*, 2013).

Public health interventions are inherently complex (Rychetnik *et al.*, 2002; Breuer *et al.*, 2015) but can act upon the social determinants of health (Macaulay *et al.*, 2017; Suchowerska *et al.*, 2019). Such complexity is a result of (i) the magnitude and the range of interacting components; (ii) the flexibility of the intervention and its fit with the local environment; (iii) behaviours, groups or levels and (iv) their outcomes (Craig *et al.*, 2008; Fletcher *et al.*, 2016; Grant and Hood, 2017). It has been suggested that social enterprises have the potential to be a ‘non-obvious actor’ in the delivery of such complex public health interventions (Roy *et al.*, 2017; Calò *et al.*, 2019), even where health is not part of the stated social mission of the organization (Macaulay *et al.*, 2017), however, more empirical evidence is needed to determine this (Calò *et al.*, 2018).

The following study is part of the CommonHealth (see https://www.commonhealth.uk/) research programme, which aimed to develop methods to evaluate new pathways to health creation and health inequalities reduction arising from community-based social enterprises. CommonHealth comprised of eight projects focussing on specific social needs/contexts including young people, rural communities (Kelly *et al.*, 2019a), homelessness (Rolfe *et al.*, 2019) and marginalized women (Hill O’Connor *et al.*, 2018). The study underpinning this paper considered social enterprises that support older people. 

In the UK, currently over one-third of the population are over 50 years (ONS, 2016), reflecting the ageing global demographic (United Nations, 2013). The number of UK residents aged over 85 years is projected to double to 3.6 million between 2015 and 2040 (ONS, 2015), putting pressure on state-delivered health and social care services as the ‘demographic deficit’ between the young economically active population and the older retired population increases (Lee and Mason, 2010; Harper, 2014). While rising health inequalities pre-date austerity, the impact of the recession on particularly vulnerable groups has focussed attention on this societal challenge. It has also positioned social enterprise as a politically and economically attractive model for the delivery of some public services, potentially increasing opportunities to generate partnerships between social enterprise and the public sector (Donaldson *et al.*, 2011; Hazenberg and Hall, 2016; Henderson *et al.*, 2019). Yet the impact of social enterprises on health and wellbeing remains poorly understood (Hlady‐Rispal and Servantie, 2018).

Researching older people is complex, not least because the term represents a heterogeneous arbitrary grouping of individuals based on chronological age which does not reflect the wide variance in ageing processes amongst its members (Kotter-Grühn *et al.*, 2016). Yet services are designed to group older individuals homogenously, dismissing the diversity of older participants’ capabilities and functioning. This can lead to mixed outcomes when attempting to establish supportive social groups regardless of other similarities, e.g. age, gender, long-term health issues (Stevens, 2001; Cattan *et al.*, 2005; Masi *et al.*, 2011). Social enterprise activities for older people are therefore challenged with delivering benefit across diverse and evolving cohorts through flexible, tailored experiences (Sen, 1999; Perissinotto and Covinsky, 2014) if they are to facilitate positive health and wellbeing outcomes (Farmer *et al.*, 2011; Steinerowski *et al.*, 2011; Kelly *et al.*, 2019b).

Physical functioning and self-efficacy are influenced by self-perceptions of ageing, demonstrating the power of psychological strategies in overcoming age-related limitations (Tovel *et al.*, 2017). Yet, the antecedents of feeling younger than one’s chronological age (i.e. subjective younger age) are poorly understood (Kotter-Grühn *et al.*, 2016). Societal stigma presents the ageing process as a negative condition which reduces status and competence (Cuddy *et al.*, 2005; Weiss and Freund, 2012; Hatzenbuehler *et al.*, 2013).

## THE DETERMINANTS OF HEALTH

**Fig. 1:**
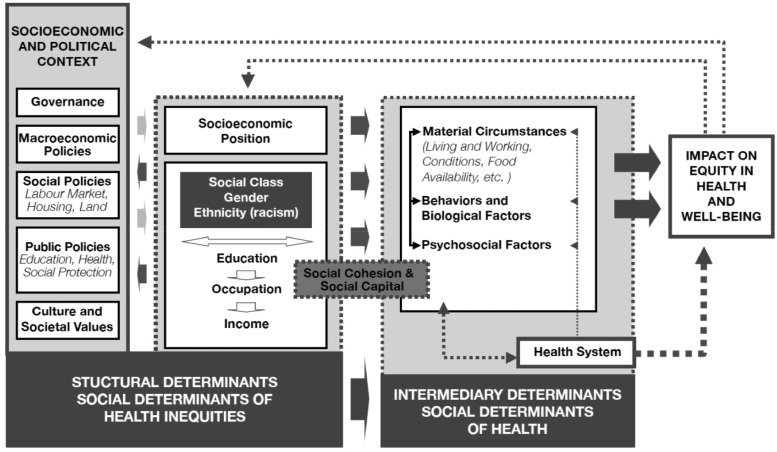
The Social Determinants of Health conceptual framework. Source: Solar and Irwin [Solar and Irwin (2010), p. 6].

The conditions in society that influence health and wellbeing are captured within the conceptual framework of the social determinants of health (Figure 1). This framework illustrates how socioeconomic and political contexts and their inherent cultural and social norms are important when combatting health inequalities and improving population health. Complex public health interventions may act on individual or multiple intermediary determinants of health to generate more equitable health and wellbeing. Should social enterprise activities be framed as complex public health interventions, their impact could therefore act upon intermediary determinants of health (i.e. material circumstances; behaviours and biological factors; and psychosocial factors) (Figure 1) to create more equitable health and wellbeing for their participants. To investigate this, the study underpinning this paper explored the impact of participation in social enterprise activities on the health and wellbeing of older people. It aimed to understand ‘what’ impact social enterprise activities have on participants’ health and wellbeing, and also ‘how’ that impact is created. The following sections explain the study context and present our partner social enterprises before describing our methodological approach. We then describe our findings in the context of current literature. We conclude with a reflection upon social enterprise as complex public health intervention that impacts upon the intermediary determinants within the social determinants of health.

## METHODOLOGY

This study specifically aimed to understand ‘what’ impact social enterprise activities had on the health and wellbeing of participants aged over 50 years [we use the EU’s active ageing policies definition that older age begins at 50 years (EPRS, 2014)], and also ‘how’ that impact was created. To fulfil these aims, this study recruited partner organizations through Social Enterprise Networks and agencies that provide local and national social enterprise support in Scotland. We identified seventeen social enterprises that aim to support older people but do not deliver clinical health and care services. We selected three organizations based on the willingness of Management/Board to participate; their delivery of recurrent activities to study; the availability and cognitive capacity of potential interviewees.

We have allocated fictitious names to our three partners to enhance participant anonymity:

1. *Invercolm*: Based in an urban area ranked as amongst the 20% most deprived in Scotland (SIMD, 2016) with at-birth life expectancy of 73 for males and 78 for females (NRS, 2017), Invercolm has traded for over 25 years and employs over 20 staff. Activities studied were an older peoples’ day centre, befriending, and a men’s group.
2. *Benaig*: Based in an urban area ranked as amongst the 10% least deprived in Scotland (SIMD, 2016) with at-birth life expectancy of 82 for males and 85 for females (NRS, 2017), Benaig employs over 60 staff and was established in 1941 but developed its social enterprise arm 12 years ago. Activities studied were an older peoples’ day centre, befriending and home care services.
3. *Lochan Languages*: Lochan operates across Scotland, and the class participating in this study were married couples (one with dementia and one as carer) from across a rural region in south west Scotland. Lochan had been operating for 1 year and had one member of staff, using sessional tutors to teach 6 weekly 2-hour foreign language classes to help older people battle dementia.

All three social enterprises delivered activities at least weekly which supported older people in community spaces. Lochan Languages, like Invercolm and Benaig, delivered an interactive activity to a group of older people who gathered specifically to attend that activity.

### Participants

This study aimed to explore the health and wellbeing impacts of involvement in social enterprise activities on all older participants regardless of their role, i.e. as staff, clients, carers or volunteers. Therefore, during March−November 2016, we sampled participants aged 50 or over who participated at least weekly in social enterprise activities. These included staff delivering the activities (*n* = 10; 51 − 73 years); volunteers (*n* = 10; 56 − 79 years); clients, e.g. service users; befriendees; customers (*n* = 18; 66 − 98 years); and clients’ carers who participated in activities (*n* = 5; 77 − 80 years).

The sample broadly reflected the characteristics of the Scottish demographic, i.e. predominantly ‘White Scottish’ with several participants from White British Other and ‘Mixed/Multiple Ethnic’ groups (NRS, 2014). Ethical approval was obtained from the University’s Ethics Committee. We consulted with staff about each individual’s ability to give informed consent prior to approaching any volunteer, client or carer. All participants were guided both verbally and visually through a hard-copy information sheet before informed consent was given to ensure auditory and/or visual impairments did not preclude participation. All individuals were told prior to agreeing to participate that we could not guarantee complete confidentiality as others who know them may recognize their story. In an attempt to enhance confidentiality, we have anonymized the organizations and individuals in the study by using fictitious names and have intentionally not stated which social enterprise our quoted participants were attending.

We developed an open-ended interview topic guide after reviewing existing measures of subjective wellbeing, quality of life and sense of coherence (Eriksson and Lindström, 2005; Barcaccia *et al.*, 2013; Vázquez *et al.*, 2015). The topic guide was used in all three social enterprises with four aims: (i) gather information about the participants’ lives; (ii) capture their involvement with the social enterprise; (iii) explore participants’ perceptions of any health impacts this involvement bestowed; (iv) gather perceptions of the social enterprise as an organization. Interviews averaged 45 minutes and were audio-recorded then transcribed.

The transcribed interviews were broadly organized under these four topic guide aims in Nvivo before we explored ‘what’ impact social enterprise activities had on the health and wellbeing of participants aged over 50, and also ‘how’ that impact was created. We used thematic analysis to achieve this by developing categories from patterns emerging within each aim through coding both deductive manifest (directly observable) themes and inductively exploring latent themes (underlying the phenomenon) (Joffe and Yardley, 2004; Braun and Clarke, 2006). We then conducted between-group and within-group analyses of these findings. All findings were discussed and reflected upon by the research team before being contextualized in the literature, giving us greater confidence in our interpretations (McKeever *et al.*, 2015).

## RESULTS

Our thematic analysis resulted in four key findings. Firstly, it found beneficial health and wellbeing impacts of involvement in social enterprise activities which demonstrated ‘what’ impact social enterprise activities had on the health and wellbeing of our participants aged over 50. Secondly, it found two antecedents facilitating perceived subjective younger age, namely ‘downward social comparison’ and ‘identity’, offering insight into ‘how’ beneficial health and wellbeing was generated by participants themselves. Thirdly, our analysis showed these two antecedents also influenced group formation. Finally, we also found evidence of situated and temporal aspects to wellbeing, highlighting the importance of context. These results were found between and within the four sampled groups (i.e. staff, clients, carers and volunteers). No between-group differences were found in types of health and wellbeing benefits.

### Health and wellbeing impact

All participants in our study reported a greater sense of purpose as a result of involvement in social enterprise activities, particularly staff and volunteers who valued the opportunity to support other people. Clients and carers emphasized the importance of having ‘somewhere to go’ each week:

…if I wasn’t coming here I'd be just sitting looking at four walls every day and I probably wouldn't even bother to get dressed. I would just be sitting in my pyjamas. (Joan, 67, client)

All participants stated they enjoyed the activities they were involved with and the time they spent with the people they met through the activities, even in the face of difficulties in achieving that participation. Isabel, a weekly volunteer who suffered from depression and agoraphobia, told us the effort to volunteer was exhausting but beneficial to her health and wellbeing:

It's difficult for me. I soon get tired…the mental fatigue that I get with being anxious all the time…I've got low self-esteem and I need to work on it…The effort has proved helpful… (Isabel, 56, volunteer)

As Isabel demonstrates, participation in social enterprise activities can require engaging in a number of difficult or challenging behaviours before and during involvement. Staff had to manage vulnerable clients, carers and volunteers, while volunteers like Isabel had to surmount significant personal challenges. Carers and clients like Joan had to organize themselves and manage significant health conditions to ensure they arrived in time to participate. The time invested in overcoming these challenges was perceived to be worthwhile, as volunteers, carers and clients reported a reduction in social isolation and an increase in social connectedness as a result of their involvement:

I think I didn't fancy meeting people. And that's not me. Because I've been in the house that long myself, I didn't realise how important it was to get out and talk to people…it really has been a great thing. (Charles, 81, client)

### Participants’ social comparisons

The social enterprises in our study created communities of people who would otherwise have never met, and this enabled participants in all roles to make social comparisons with others outside their normal social contacts. However, our data showed they only made comparisons with other participants on dimensions where the outcome would be positive for them. Wills (Wills, 1981) built on Festinger’s (Festinger, 1954) Social Comparison Theory to identify this mechanism as ‘downward social comparison’, i.e. individuals compare themselves or their situation with others who are less fortunate than themselves. Such downward social comparisons are self-protective when someone is presented with negative information about their age (Weiss and Freund, 2012). We found examples of this amongst older people in each role within our sample, like Duncan, who stated he had ‘a clear mind’ with ‘no dementia or anything like that’. He commented:

…they help a lot of people that’s handicapped. The people with chairs and sticks and all that…Fortunately enough, I can still walk a short distance. (Duncan, 86, client)

Duncan’s downward social comparison focussed positively on his remaining capabilities and functioning, rather than on deficits like his age-related blindness. Jessie, a staff member, focussed on how good her life circumstances were in comparison to the clients she met:

It sounds a bit like I’m comparing myself to them but I’m not. But it just lets me see how well off I am in reality to some of the other people that we do go and visit… (Jessie, 59, staff)

Although Jessie implied she did not want to make a comparison between herself and the clients she assisted, we found participants like Jessie obtained individualized beneficial outcomes (enhanced feelings of wellbeing) through their selective use of downward comparative dimensions.

### Participants’ identities

Older people have been found to protect their wellbeing by distancing themselves from others who are the same chronological age, hence reducing self-internalized ageism which could threaten their wellbeing (Weiss and Freund, 2012). Widespread societal perceptions present older people as incompetent and of low status (Levy, 2003; Cuddy *et al.*, 2005; Weiss and Freund, 2012) and as ‘the elderly stereotype is widespread and resistant to change…it is difficult for an individual elderly person to override’ [Cuddy *et al.* (2005), p. 276]. Given this, it is perhaps unsurprising we found no-one directly described themselves as ‘old’ in our study. When someone realizes they are socially identified as ‘old’, they become vulnerable to negative self-perceptions which further reinforce their internal negativity towards ageing (Shenkin *et al.*, 2014). We found some clients like Evelyn exhibited this pattern. She suffered an acute health crisis leaving her permanently dependent upon a tri-walker, a physical symbol of age-related decline. Evelyn struggled with this unexpected transition in identity as she changed from being an active volunteer in her community to suddenly being vulnerable and dependent. She became voluntarily housebound:

I wasn’t going out with that *(the tri-walker)* because people look at me. The neighbours will be saying, ‘Oh, poor Evelyn’…I didn’t want anybody feeling sorry for me. (Evelyn, 72, client)

She was reluctantly persuaded to attend one of the participating social enterprises’ day centres after months of not leaving home. She developed a strategy to redefine the day centre activities, however, which enabled her to maintain her subjective younger age despite attending what she previously labelled an ‘old people’ activity:

I used to say that, you know, ‘This is only for old people’. But it’s not really. It’s good! (Evelyn, 72, client)

Similarly, when asked about her participation in day centre activities, Theresa, a client, stated:

You listen to old people’s life stories. I mean, I like reading. So therefore I like listening to people’s life stories. (Theresa, 85, client)

By describing herself as passively listening to ‘old people’, Theresa distances herself from her groupmates (Weiss and Freund, 2012) and redefines her role within the activity to maintain her subjective younger age, i.e. she is not ‘old people’. Yet she was amongst the chronologically oldest attending that activity.

This phenomenon is explained within Self-Categorization Theory (Turner *et al.*, 1987) which suggests social identity is the internal cognitive mechanism that facilitates group behaviour and enables us to categorize ourselves as sharing identity with a group (i.e. ‘us’/’we’) or not (i.e. ‘them’). This shared social identity has been identified as a beneficial causal mechanism facilitating positive impacts on health and wellbeing amongst older people (Gleibs *et al.*, 2011a,b; Haslam *et al.*, 2014). We found some participants across all roles in our sample demonstrated this shared identity mechanism through their use of ‘us’ and ‘them’, like Marilyn, a day centre volunteer:

…I actually quite enjoyed old people’s company. I actually found…I could talk to them. I could play games with them…I found I got on really well with these old people. (Marilyn, 70, volunteer)

While Marilyn distanced her social identity from clients, other volunteers and clients formed close relationships with each other. For example, two befrienders, Joyce (a retiree) and Thomas (unemployed) were both socially isolated themselves. Both formed close relationship with those they befriended:

…we still managed to get about, and then his eyesight deteriorated, so we just had to sit in in the last year of his life…He died a year past January this year. But oh, it’s broke my heart. I was really close to him. (Joyce, 76, volunteer)

Thomas spent a lot of time with Jimmy, his befriendee:

…we went shopping. Jimmy loves shopping. He just loves shopping. And we went to *(supermarket1)*, then we went to *(supermarket2)*, and then we went to *(supermarket3)* for all his shopping. And he made me my dinner….his chilli’s lovely. (Thomas, 60, volunteer)

All befrienders reported they enjoyed and valued befriending, but only some reported mutually beneficial relationships. How they achieved value and enjoyment differed depending on participants’ own needs and perceptions. Joyce and Thomas shared social identities with their befriendees which facilitated their friendships. Shirley also enjoyed and valued befriending, but distanced herself from befriendees using both identity strategies and downward social comparison:

…although I do have friends and family, I just feel these people are on their own…you just feel so sorry for them…they are in their house each and every day sitting on their own…you just think ‘What kind of life have they got?’ (Shirley, 59, volunteer)

Our study found that participation in social enterprise activities resulted in some individuals having to confront their chronological age and ageing processes. However, participants bolstered their subjective younger age using social identity and downward social comparison mechanisms. The social contact created during social enterprise activities afforded the opportunity for individuals to employ these mechanisms to enhance their wellbeing.

### Group formation

We found social identity influenced the successful formation of groups. The relationship between group membership and improved health is longstanding and well documented (Campbell and Jovchelovitch, 2000; Jetten *et al.*, 2014), yet quality social connections can be difficult to manufacture even when members are lonely and/or isolated (Stevens, 2001). More understanding is therefore needed about why some interventions are more successful than others (Cattan and White, 1998; Cattan *et al.*, 2005; Masi *et al.*, 2011), and our evidence allowed us to explore this.

As reported earlier, Marilyn and Theresa distanced themselves from the social enterprise-generated groups they participated in. However, some activities in our study presented opportunities for individuals to form into smaller supportive groups which shared a social identity, such as the carers who brought their partners to a Lochan Languages course. They reported feeling socially isolated before they began the classes. For example, Audrey was coming to terms with the progression of her husband’s dementia and with her new identity as his carer. She reported she was becoming increasing isolated because her friends did not understand the daily challenges she faced. However through her husband’s participation in Lochan Languages, she had met other carers:

…you realise that what’s happening to their husbands is happening to my husband, and we just talk about these things…how you cope… (Audrey 77, carer)

Yvonne, another carer in the group, spoke of the groups’ connection:

…all of the people here…understand the situation. They know what you’re going through. They *(the dementia sufferers)* are similar in so many ways. (Yvonne, 80, carer)

Audrey, Yvonne and the other carers in the group were navigating their changing life circumstances together through their shared social identity. The group continued to meet even after the block of classes had ended, creating a new self-managed carers’ support group that was highly valued by its members.

Invercolm’s Men’s Group enabled men to socialize together while participating in games. The men self-grouped across three tables and most chose to play dominoes every week. Two of the three tables of men participating in this activity did not develop deeper friendships, nor did they report relying upon the other table members for social support. However, the third table located at the top of the hall (the ‘top’ table) reported a particularly strong social connection with each other:

All your pals are here. The same crowd of us play at that table every week. Other ones want to come and play at our table but we don’t let them. No. This is the top table, you don’t get to play here! (laughs). (Douglas, 80, client)

Douglas’ perception of the friendship he shared with the men at the top table was echoed by other top table members, like Hamish:

…there’s six guys at that table and we’ve all got our own problems…all get on well together…I couldn’t tell you the names of the other guys on the other tables (laughs). (Hamish, 69, client)

As Hamish implied, the men at the top table shared their problems together, including talking openly about the loss of their wives. Douglas’ wife had passed away, while Hamish’s wife had been admitted to a residential care home. Just as the carers earlier had reported feeling less socially isolated as a result of the language class, these men shared their life changes and challenges with each other each week as they came to terms with living alone. Alan was another top table member who had lost his wife. He reported struggling with depression and valued the other men’s support:

I kept telling the boys that. I kept telling them that. ‘If it weren’t for you men in here, I probably wouldn’t be here today’. (Alan, 78, client)

The success of manufactured groups has proved variable (Cattan *et al.*, 2005; Masi *et al.*, 2011), and we found this to be true in our study. The Lochan carers and the top table men were very supportive of each other and shared a bond they valued. However, other groups in our study did not appear to share their social identity with each other, despite also reporting they enjoyed the activities and valued the company of others they met as a result of their social enterprise involvement.

## DISCUSSION

Our study aimed to understand ‘what’ impact social enterprise activities had on the health and wellbeing of participants aged 50 and over, and also ‘how’ that impact was created. While our study was small and exploratory, and hence our findings are suggestive rather than conclusive, we found the impact of involvement in social enterprise activities was indeed beneficial to health and wellbeing, and increased participants’ sense of purpose, social support, connectedness and inclusion. Our findings address a gap in current knowledge through this insight into older people’s experience of participating in social enterprise activities (Hlady‐Rispal and Servantie, 2018).

We also presented evidence explaining ‘how’ some health and wellbeing benefits were generated. We found two mechanisms employed to mitigate negative self-perceptions of being involved in activities labelled as ‘for older people’ and preserve subjective younger age, namely downward social comparison and identity management strategies. As knowledge about the antecedents of subject younger age is currently lacking (Kotter-Grühn *et al.*, 2016), our evidence provides important new insights.

### Social enterprise as a complex public health intervention

**Fig. 2:**
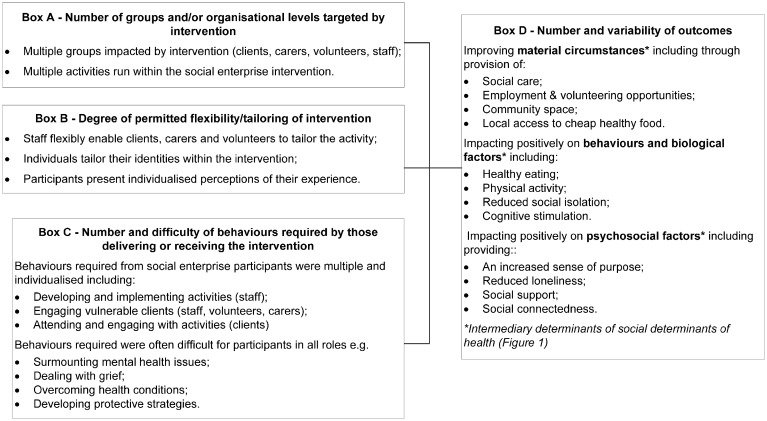
Social enterprise as a complex public health intervention. Derived from Craig *et al.* (Craig *et al.*, 2008).

‘What’ impact social enterprise activities had on the health and wellbeing of participants can establish whether those activities can be considered as public health interventions. Craig *et al.* (Craig *et al.*, 2008) propose that complex public health interventions can be so-defined if their activities fulfil a number of dimensions. Figure 2 presents a framework describing how the social enterprise activities we studied could be considered as complex public health interventions under Craig *et al.*’s dimensions.

The number and variability of outcomes individuals received from participation (Figure 2, Box D) reflects the complexity and multilevel impact of the social enterprise activities we studied. We conclude therefore that our exploratory study presents evidence that social enterprise activities could be considered as a complex public health intervention by fulfilling Craig *et al.*’s (Craig *et al.*, 2008) definition.

In Figure 2, Box D, we also describe how the health and wellbeing outcomes we found directly impact upon intermediary social determinants of health (Figure 1). Furthermore, our study found that participation in social enterprise activities impacted upon the socioeconomic position of some participants through providing employment and generating vocational training, demonstrating that involvement in social enterprise can also impact positively upon the structural determinants of health (Figure 1). Our findings therefore support the suggestion that social enterprise acts upon a range of vulnerabilities (Haugh and Kitson, 2007; Donaldson *et al.*, 2014) by providing access to social care, increasing the social connectedness of participants, and reducing social isolation amongst marginalised older people.

While offering currently lacking insight into the impact of social enterprises on health and wellbeing (Hlady‐Rispal and Servantie, 2018), our small exploratory study was limited to three social enterprises located across the central belt of Scotland. We recruited a sample of individuals already participating in these activities. Although our sample reflected the demographic structure of the Scottish population, the majority of these individuals were White Scottish and hence the sample’s diversity was limited.

## CONCLUSION

The conceptual framework of the social determinants of health presented in Figure 1 illustrates the importance of socioeconomic and political contexts when combatting health inequalities. Socioeconomic changes, including an increasing older demographic (United Nations, 2013) and the impact of the austerity agenda, are forcing cuts to public spending and a transformation of existing services at a time of increasing need (Hazenberg and Hall, 2016). In the UK, a supportive social enterprise policy environment (Steiner and Teasdale, 2017) enhances social enterprise’s potential to deliver future health and care service provision that could mitigate some of the impending socioeconomic difficulty in caring for a growing older population. Considered as a complex health intervention, social enterprises can contribute to the health and wellbeing of those disadvantaged, hard to reach and marginalized in our society. As these groups of the population are difficult (and expensive) to target through traditional public intervention, the findings from this exploratory study suggest social enterprises could play an important role in addressing current and forthcoming health and wellbeing service provision challenges.

## ETHICAL APPROVAL

This research received ethical approval from Glasgow Caledonian University's GSBS Ethics Committee.
